# 36 Months’ Clinical Performance of Primary Incisors Restorations Depending on the Type of Restorative Technique Used: A Randomized Controlled Trial

**DOI:** 10.3390/dj9110126

**Published:** 2021-10-22

**Authors:** Maria Sarapultseva, Alexey Sarapultsev

**Affiliations:** 1 Medical Firm Vital EBB, 136 Sheinkmana Str., 620144 Ekaterinburg, Russia; 2 Institute of Immunology and Physiology (IIP), Ural Branch of Russian Academy of Sciences, 106 Pervomayskaya Str., 620049 Ekaterinburg, Russia; a.sarapultsev@gmail.com

**Keywords:** caries risk, composite layering technique, early childhood caries, pediatric dentistry restorations, primary teeth caries, strip crowns

## Abstract

Background: Depending on the stage of the disease and the child’s age, different types of interventions can be used to treat early childhood caries. As a result, there is not enough clinical evidence to show that one kind of restoration is better than another. The objective of this longitudinal study was to compare the results of 36 months of clinical performance of primary incisors restorations using an incremental layering technique with the ceram.x^®^ SphereTECTM nanoceramic composite (Dentsply) or a full coverage technique with transparent strip crowns (Frasaco GmbH) with the same composite in children with or without biological caries risk factors. Methods: 80 patients (females 42/52.5%) were included in the study. A total of 160 restorations were performed. Restorations were evaluated at baseline and at 6, 12, 24, and 36 months, according to modified Ryge criteria. Conclusion: Restorations with both techniques were clinically highly successful and showed similar clinical performance at postoperatively regardless of the presence of biological factors of caries risk.

## 1. Introduction

The presence of one or more decaying (non-cavitated or cavitated lesions), missing (due to caries), or filled tooth surfaces in any primary tooth in a preschool-age child under the age of 71 months is classified as early childhood caries (ECC) [1,2,3,4,5,6]. ECC is one of the most common childhood disorders, affecting millions of children worldwide [1,2,3,4,5,6,7,8]. According to recent studies, ECC prevalence in the world ranges from 25% to 90% [3], with a mean ECC prevalence of 23.8 percent in infants younger than 36 months and 57.3 percent in children aged 36 to 71 months, respectively [1,2]. ECC in developing countries was reported to be more than in developed countries, depending on the availability of universal health coverage, economics (e.g., gross national income values), and cultural traditions [1,2,6,7,8,9,10,11]. While the prevalence of ECC is between 2 and 19 percent in most developed nations [1,7,8,11], it has been estimated to be as high as 58–90 percent in less developed countries and among disadvantaged populations in industrialized countries [4,5,6,7,8,9,10,11,12,13].

The prevalence of ECC in Russia ranged from 83 to 93.4 percent, with mean DMFT values (decayed, missing, or filled teeth) of 3.0–6.71 [10,14,15]. At the same time, even in developed nations, ECC prevalence does not appear to be decreasing: in Germany, it exceeds 10% (up to 26% with early lesions) among 3-year-old children and escalates to almost 50% in 6-/7-year-old children [11,16,17]. Furthermore, whereas the DMFT-index has declined over time in general, the prevalence of ECC has not, and recent studies revealed the increasing ECC burden [8,16,17]. Therefore, global data suggest that ECC is still very common but rarely treated [1,3,4]. As a result of the pronounced damage and subsequent loss of teeth in the absence of appropriate treatment, major functional and aesthetic issues emerge [8]. That is why pediatric dentistry places such a high value on the proper repair of central incisors in children.

Conventional treatment techniques range from fluoride application to complete coverage with crowns [4,18,19,20,21,22,23]. The use of standard or custom-made crowns on the front group of primary teeth is one of the most commonly used restoration procedures for ECC. This procedure is predictable and enables a long-term aesthetic and functional outcome [20,23,24]. At the same time, the materials used to make crowns (such as zirconium di-oxide or metal crowns veneered with acrylic) necessitate very thorough tooth preparation, which raises the risk of unintentional pulp chamber perforation. Furthermore, restoring teeth with crowns made of zirconium dioxide or metal veneered with acrylic does not allow correcting the restorations in case of failure [25,26]. Pedo Jacket crowns require less harsh preparation and have a simpler construction. Still, their relatively high cost and the presence of legislative restrictions on their usage in certain countries severely limit their use [27]. Due to the small size of primary teeth, the proximity of pulp to the tooth surface, and the comparatively thin enamel and surface area for bonding, esthetic restorations in children are always challenging [28]. Moreover, low dentine mineralization, a thin hybrid layer that bonding agents do not thoroughly permeate, and a prismless layer of a primary tooth that does not respond well to acid etching contribute to restorative failure [28].

However, there has been a shift to more conservative preparations and restorations as the usage of novel adhesive restorative materials, and bonding technologies has risen [4,22,24,29,30]. Nowadays, both resin-based composite materials and resin-modified glass ionomer cements are considered appropriate for anterior teeth, according to the AAPD Guidelines (2019), depending on the possibility of proper isolation [31]. Incremental layering, bulk-fill, and full-coverage with transparent celluloid caps-strip crowns are recommended application techniques for these materials [18,22,23,29,30,31,32,33]. According to several studies, the type of restoration technique used may be determined by the individual’s risk of acquiring caries [19,20].

According to the AAPD Caries Risk Assessment Guideline [18], the presence of one or more carious surfaces places the child in a high-risk group. However, caries-risk assessment models contain so-called biological elements, which may have an extra impact on dental health and restoration durability. With that, there are no comparative studies of temporary teeth restorations performed using the classical layering technique and strip crowns depending on the caries risk or factors associated with the caries risk with a follow-up period of more than 24 months. That is why this study was aimed to compare the results of 36 months’ clinical performance of central incisors restorations in primary dentition depending on the type of restorative technique used and the presence or absence of biological factors of caries risk. The null hypotheses tested were as follows: (1) the long-term results of restorative treatment would not depend on the presence or absence of biological factors of caries risk. (2) There would be no differences between the tested restorative techniques in terms of longevity [34].

## 2. Materials and Methods

### 2.1. Study Design

The prospective randomized controlled trial was conducted at the pediatric department of the private dental clinic Vital EBB (Ekaterinburg, RF).

Ethical approval was obtained from the Institute of Immunology and Physiology (IIP) of the Ural Division of Russia Academy of Science, Ekaterinburg (14 April 2017, D-16-4-2017), and informed consent was obtained from all parents or legal guardians of subjects recruited for the study. Trial registration: Retrospectively registered in ISRCTN Register (London, UK) with ISRCTN64028277. The registration was made on 11 August 2021.

Families were given enough time to consider the information, had any questions answered, and provided their agreement freely and voluntarily. Patient consent forms, incorporating Guidelines of Federal Compulsory Medical Insurance Fund of Russian Federation (1999 # 5470/30-Z/i) and ADA Principles of Ethics and Code of Professional Conduct, were distributed to parents at reception areas of the dental clinic (ADA 2012). Requested information included name and date of birth of pediatric patient; name, relationship to the patient, and legal basis for an adult to consent on behalf of a minor; description of specific treatment undertaken (in simple terms); alternatives to treatment; potential complications of the treatment; acknowledgment by a patient or parent/guardian that all questions were answered; and signed by a dentist, parent or legal guardian, and witness.

### 2.2. Inclusion and Exclusion Criteria

Pediatric dental patients were included in the research after informed consent was obtained from all parents or legal guardians of subjects recruited for the study. The presence of two Class-four caries cavities on the central incisors was the clinical inclusion criterion. This study did not include patients having a history of coronary artery disease, pre-excitation syndromes, motor impairments (cerebral palsy and epilepsy), pacemakers, drug users, or dialysis patients. Patients whose parents or guardians refused to give their consent to participate in this study were also excluded. Patients with teeth with coronal defects resulting from trauma were excluded from the study.

### 2.3. Study Population

The study included 80 high caries-risk patients (males 38/47.5%; females 42/52.5%) with two class IV cavities in primary central incisors. The mean age of the patients was 30 months.

### 2.4. Study Interventions

Eighty-four patients were chosen for the study after the inclusion and exclusion criteria were applied. Four patients were removed from the trial because they refused to participate. The patients were given a complete clinical evaluation after being chosen for the trial. They were allocated to one of two pediatric dentists at random by the research coordinator. Every single case was examined and treated by each operator. Caries risk was assessed using a national protocol and the AAPD Guideline on caries-risk assessment [18] by trained and calibrated dentist examiners at each clinical examination (intraexaminer kappa coefficient value > 0.82). The examination included the following components: (1) medical and dental history; (2) examination of the maxillofacial area, oral cavity, dentition, and soft tissues; (3) radiographic assessment of hard teeth and periapical tissues (periapical X-ray in all cases); (4) assessment of caries risk [18,19].

Possible abnormalities of the crown and root structures, as well as the surrounding bone, were detected using intraoral radiography. Every patient underwent a periapical X-ray with a #1 or #0 size Phosphorous Image plate (PIP) at maxillary central-lateral projection. In all of the instances, periapical X-rays were taken using the paralleling technique. To obtain the greatest possible PIP placement, the film holder was employed. The X-ray examination in this study was performed using Duerr Dental AG’s PIP and film holders, as well as the film scanner “VistaScan Mini”.

### 2.5. Treatment Strategy

Patients were equally divided into two main groups according to the presence of biological factors of caries risk according to the AAPD Guideline [19]. Group 1 comprised 40 patients without any of the biological factors of caries risk (mother/primary caregiver has active cavities; parent/caregiver has low socioeconomic status; a child has >3 be-tween-meal sugar-containing snacks or beverages per day; a child is put to bed with a bottle containing natural or added sugar; a child has special health care needs; a child is a recent immigrant). Group 2 included 40 patients with the biological factors of caries risk.

**Figure 1:**
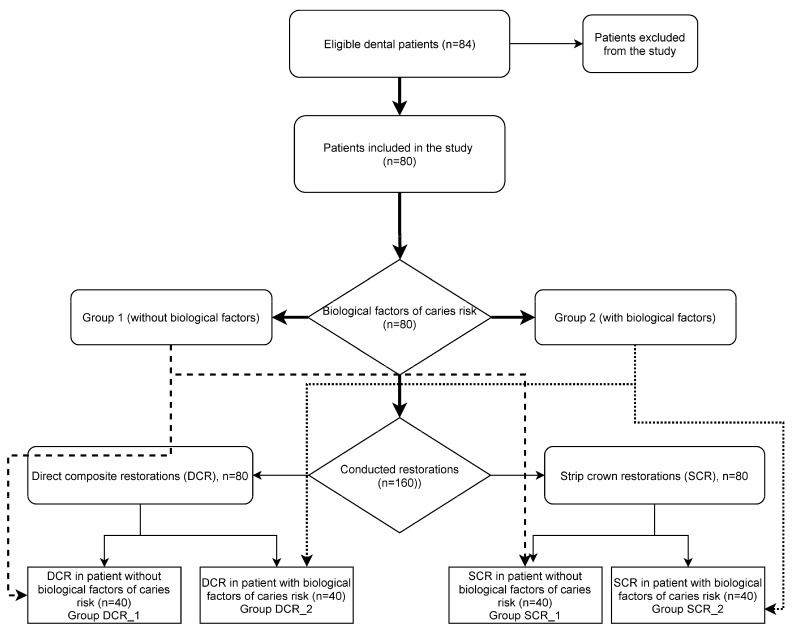
Flow diagram for study participants and study interventions.

**Figure 2:**
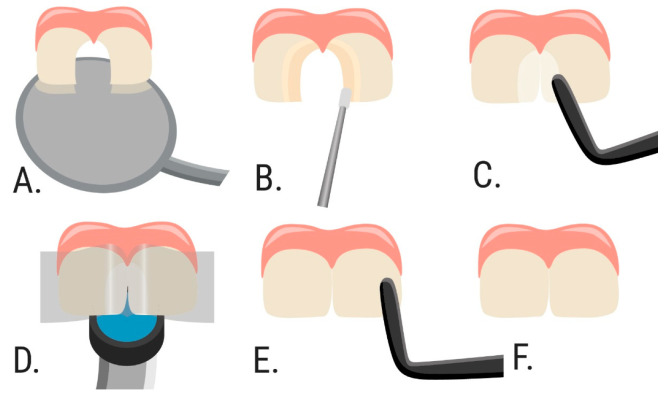
Schematic layout of class IV cavities restoration with an incremental layering technique, implemented in the research. (**A**). Step 1—prepared class IV cavities on two central incisors. (**B**). Step 2—applying the adhesive system. (**C**). Step 3—the first increment of the composite is restoring a palatal enamel. (**D**). Step 4—the second increment of the composite material is restoring the contact wall; the transparent polyester strips are placed to separate two restorations and create medial walls. (**E**). Step 5—the third (final) layer of the composite material is restoring the vestibular part of a tooth. (**F**). Step 6—restored cavities at both central incisors (final result).

**Figure 3:**
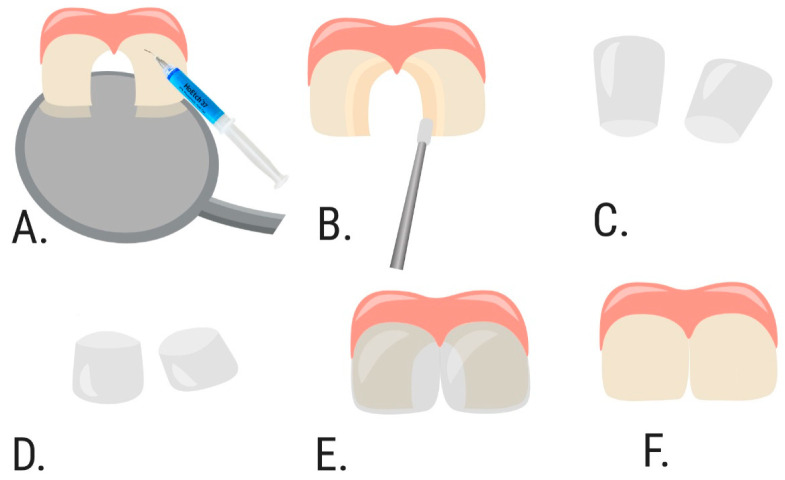
Schematic layout of class IV cavities restoration with strip crowns and composite material. (**A**). Step 1—prepared class IV cavities on two central incisors, application of 37% phosphoric acid to the entire enamel surface (both vestibular and palatal). (**B**). Step 2—applying the adhesive system. (**C**). Step 3—choosing the appropriate size of strip crowns. (**D**). Step 4—prepared strip crowns (cut to a height). (**E**). Step 5—strip crowns are placed to the teeth. (**F**). Step 6—restored cavities at both central incisors (final result).

The patients from those Groups (1 and 2) were randomly divided into two subgroups according to the restoration techniques (Figure 1). The treatment plan describing the restorative technique was given to the operator by the research coordinator. In the DCR group (80 restorations), cavities were restored by direct composite restoration with an incremental layering technique (Figure 2), in the SCR group (80 restorations)—with full coverage technique with transparent strip crowns (Figure 3). Thus, four groups (DCR_1 and DCR_2, and SCR_1 and SCR_2) with 40 restorations in each were evaluated.

Local anesthesia with 3% Mepivacaine solution (plain) was used. The dosage of the anesthetic did not exceed the maximum recommended amount of 4.4 mg/kg.

Cavities were prepared with the high-speed turbine burs under copious water cooling at the enamel level, and with the low-speed carbides burs and hand excavator at the dentin level. The conservative tooth preparation was implemented in all the cases (there was no extra retentive preparation done, such as boxes, groves, etc., only a small enamel bevel was prepared to removed unsupported enamel prisms). The prepared cavities were disinfected with 1% chlorhexidine solution. The operative field isolation was conducted by a rubber dam fixed by cords and floss to make the procedure atraumatic and comfortable for pediatric patients.

The selective enamel etching technique and self-etch adhesive system were used in all the subgroups. Additionally, 37% phosphoric acid gel (Vococid, VOCO) was applied on enamel for 20 s and after copious rinsing for 40 s and indirect drying with water/air syringe the bonding agent Prime and bond NT (Dentsply) was applied.

The class IV cavities of DCR groups (DCR_1 and DCR_2) were restored with the incremental layering technique using the nano-ceramic composite ceram.x^®^ SphereTEC™ one (Dentsply, York, PA, USA). The same composite material was used to restore the cavities in subgroups SCR groups (SCR_1 and SCR_2) using the full-coverage technique with transparent strip crowns (Frasaco GmbH, Tettnang, Germany). The finishing adjustment was made with the Sof-Lex™ Contouring and Polishing Discs (3M, Brownwood, TX, USA), and final polishing was conducted with the Enhance Finishing and PoGo Polishing systems (Dentsply).

### 2.6. Evaluation of the Restorations

At baseline, 12, 24, and 36 months the restorations were evaluated independently visually with mirror and probe according to the modified Ryge criteria (Table 1) by two experienced dentists who were not involved with the insertion of the restorations. The cavosurface marginal discoloration and color match were evaluated visually after air-drying the tooth and after removing the plaque (if necessary). Before the study, these rating dentists were calibrated by a joint examination of direct composite restorations (kappa coefficient value > 0.81). When there was disagreement during the evaluation, the ultimate decision was made by forces consensus.

### 2.7. Data Analysis

Data management and analysis were carried out using R 3.1.1 software 12 (R Foundation for Statistical Computing, Vienna, Austria) and Microsoft Excel version 14.0. The statistical analysis used the Kaplan–Mayer survival model to describe the long-term results of dental procedures and a log-rank test to compare them. Any transition from the A (Alpha) criterion in the Ryge’s rating scale was considered an event in the calculations. If the transition from criterion A (Alpha) did not occur for 36 months, then these observations were censored. The calculations were performed for each of the parameters (Color match, Cavosurface marginal discoloration, Marginal integrity, Surface texture, Postoperative sensitivity, Secondary caries, and Fracture) separately.

## 3. Results

This study evaluated 160 restorative procedures carried out on 80 patients. The null hypothesis consisted of the assumptions that the long-term results of restorative treatment would not depend on the presence or absence of biological factors of caries risk, and there would be no differences between the tested restorative techniques in terms of longevity [35]. The results failed to reject the null hypothesis.

According to the results (Table 1), the success rate of restorations in subgroup DCR_1 was 94%. By the 36th month, four cases of Color match deterioration, four cases of cavosurface marginal discoloration, two of Marginal integrity, three of Surface texture, two of Secondary caries, and one of Fracture were detected. Postoperative sensitivity was not observed.

The success rate of restorations in subgroup DCR_2 was 90%. By the 36th month, five cases were observed in Cavosurface marginal discoloration and Marginal integrity, four in Color match, Surface texture, Secondary caries, Fracture. Postoperative sensitivity was detected in two cases.

The success rate of restorations in subgroup SCR_1 was 97%. At the end of the observation period, one case of Color match deterioration, Cavosurface marginal discoloration, secondary caries and Fracture, two cases each in Marginal integrity, and Surface texture were observed. Postoperative sensitivity was not detected.

The success rate of restorations in subgroup SCR_2 was 96%. By month 36, there was one case of Color match, Cavosurface marginal discoloration, Postoperative sensitivity, Secondary caries, and Fracture. There were three cases in Marginal integrity and Surface texture. The comparison of long-term results of direct composite restoration (DCR) between children with the presence or absence of biological factors of caries risk has revealed that the average probability of survival for all parameters in Group 1 was 0.943, while in Group 2—0.9; the average survival time for those parameters in Group 1 was 35.4 months, and in Group 2—35.3 months (Table 2).

According to the results of the log-rank test, there were no statistically significant differences in survival between those groups in any of the estimated parameters, as shown in Table 3.

The comparison of long-term results of strip crown restorations (SCR) in children with and without biological factors of caries risk has revealed that the average probability of survival for all parameters in Group 1 was 0.971, while in Group 2—0.961; the average survival time for those parameters was equal (35.9 months) in both groups (Table 2).

According to the results of the log-rank test, there were no statistically significant differences in survival of any parameter between those groups in any of the parameters, as shown in Table 4.

The additional calculations within groups of children with and without the biological factor of caries risk were also performed. The survival rate comparison between two types of restoration within Group 1 revealed no statistically significant differences (Table 2 and Table 5). Similar results were obtained in the analysis of survival in Group 2 (Table 2 and Table 6).

## 4. Discussion

Depending on the stage of the disease and the child’s age, different types of interventions can be used to treat ECC [4,21,22,23,29,30,35,36,37,38,39,40]. Thus, full-coverage crowns can be employed in cases of substantial tooth structure loss owing to caries, whereas occlusal, Class II, Class III, and Class V restorations can be performed with glass ionomer or resin-modified glass ionomer cement, or resin-based composites [4,21,22,23]. Among them, resin-based composites have a stronger bond and compressive strength than glass ionomer cement.

The present study results have revealed the high success rates of composite strip crowns restorations equal to 96–97% in 36 months [28]. This finding is consistent with Kupietzky et al. (2003), who demonstrated the considerably high success rate of res-in-bonded composite strip crowns restorations—88 percent with an 18-month follow-up period [37]. Likewise, Ram and Fuks (2006) reported a similar percentage of clinically effective resin-bonded composite strip crowns (almost 80%) in their 24-month follow-up study [38]. In the latter case, the retention rate was lower in teeth with three or more decay surfaces, especially in children with a high caries risk [19,20,36]. Subsequently, several studies [37,38,39,40] have recommended using full-coverage procedures in children with an in-creased risk of caries development. By contrast, Walia et al. (2014) reported lower retention qualities of strip crowns compared to zirconia crowns and veneered steel crowns during 6 months of follow-up [41]. With that, the success of composite restorations is directly de-pendent on the quality of the implementation technique, isolation of the operating field, and material selection, which, in the case of non-compliance with the thickness of the layers or incorrect polymerization, can give significant polymerization shrinkage [27,35,36]. Thus, the discrepancy in the reported success rates of restorations could partly be attributed to the high sensitivity to technology [27,35,36,37,38,39,40,41,42].

Several studies found a link between caries experience (which is considered one of the “clinical factors” for caries risk) and the rates of restoration failure [4,5,19,20,41]. On the other hand, socioeconomic status, maternal caries experience, parental education, oral health knowledge, and illiteracy, as well as a variety of child-rearing behaviors such as prolonged night-time breastfeeding and diet features (all of which are considered “biological factors”), have all been linked to ECC in children [5,43,44]. With that, there is little data available on the influence of caries-risk factors on the survival of restorations in the literature. The parent’s socioeconomic situation, educational level [45], dietary habits [46], and the existence of systemic disorders [47] have all been demonstrated to influence the durability of posterior restorations. However, this study has not shown statistically significant differences in the anterior restoration’s success rate among patients with or without biological factors. This inconsistency may be due to the discrepancies in the research methodology (employing the teeth that underwent pulp intervention) and the quality of execution techniques as evidenced by the relatively low rates of restorations survival (about 57.9–65.2% at 36 months follow-up with an overall annual failure rate of 16.7–20%) [19,20,41].

The clinical performance of composite, strip crowns, biological restoration, and composite with stainless steel band during nine months was compared in a study by H Duhan et al. (2015) [48]. According to the authors, biological restoration was determined to be the most pleasing aesthetically due to color harmony with the patient’s teeth [48]. With that, the 18-month follow-up study of XX Chen revealed that strip crowns performed well for restoring primary incisors with large or multisurface caries [49]. In the present study, however, no statistically significant differences in the clinical performance of the various restorations within the groups were observed. This discrepancy in the results can be explained by the fact that the previous studies were heterogeneous and included both teeth affected by caries and trauma [41,50], which could affect the stability of the restorations.

In summary, it has been shown that the long-term results of ECC’s restorative treatment in patients at high caries risk did not depend on the technique used or the presence of biological factors of caries risk. With that, dentist factors such as age, country of qualification, and employment status, have also been found to influence the survival of restorations. Thus, a long survival can be expected only if a patient, operator, and materials factors are considered [51,52,53].

The present study has several limitations. First, because all data were collected from patients of a single dental clinic in Ekaterinburg, Russia, the study is prone to selection bias and sample error. The sample size has been calculated to achieve a power of 0.8 in each subgroup (DCR_Group1, SCR_Group1, DCR_Group2, SCR_Group2). The required number of cases was calculated separately for each criterion in a subset (Table 2, Table 3, Table 4 and Table 5). Un-fortunately, the estimated sample size was not reached in any case due to too small differences between subgroups [54]. Therefore, the study’s results should be verified with studies conducted on a representative sample of the population. Second, the study might be subjected to selection bias concerning the patient enrollment, as the study participants appeared to be reliable and motivated regarding regular attendance and oral hygiene were chosen. Finally, dentist (age, country of qualification, and employment status) and patient (the type of patient’s arch) factors have been found to influence the survival of directly placed restorations but were not evaluated in the present study.

Therefore, the results should be interpreted cautiously, and a more in-depth analysis of factors contributing to the caries risk factor’s impact on the restoration’s performance is needed.

## 5. Conclusions

In this study sample, the primary incisor restorations conducted with the incremental layering technique using the nanoceramic composite and the full-coverage technique with transparent strip crowns with the same composite were highly successful clinically. They showed similar clinical performance at 6, 12, 24, and 36 months postoperatively regardless of the presence of biological factors of caries risk. Thus, in a clinical setting, considering the results of the present study, the best decision regarding caries management via incremental layering or the full-coverage technique is to be made by the dentist, recognizing individuals’ differences and preferences. The current findings support the scientific evidence for a reliable comparison of the two restorative techniques.

## Figures and Tables

**Table 1:** The modified Ryge criteria used in the study.

Characteristic	Rating	Criteria
Color match	Alpha Bravo Charlie	Restoration matches adjacent tooth structure in color and translucency Mismatch in within an acceptable range of tooth color and translucency Mismatch is outside the acceptable range
Cavosurface marginal discoloration	Alpha Bravo Charlie	Absence of marginal discoloration Presence of marginal discoloration limited and not extended Evident marginal discoloration penetrated toward the pulp chamber
Marginal integrity	Alpha Bravo Charlie	Closely adapted no visible crevice Visible crevice, explorer will penetrate Crevice in which dentin is exposed
Surface texture	Alpha Bravo Charlie	Smooth surface Slightly rough or pitted can be refinished Rough, cannot be refinished
Postoperative sensitivity	Alpha Bravo	Absence of dentinal hypersensitivity Presence of dentinal hypersensitivity
Secondary caries	Alpha Bravo	No evidence of caries Caries is evident
Fracture	Alpha Bravo	No evidence of fracture Evidence of fracture

**Table 2:** Descriptive survival statistics based on the Kaplan–Mayer survival model.

Group	Criterion	Type of Procedure	Survival Probability	Number of Events, %	95% CI Lower	95% CI Upper	R	Mean Survival
Group 1	CM	DCR	0.900	10.0	0.812	0.998	0.29	34.8
		SCR	0.975	2.5	0.928	1.000	3.51	36.0
	CMD	DCR	0.900	10.0	0.812	0.998	0.29	34.8
		SCR	0.975	2.5	0.928	1.000	3.47	35.7
	MI	DCR	0.950	5.0	0.885	1.000	1.00	35.7
		SCR	0.950	5.0	0.885	1.000	1.00	35.7
	ST	DCR	0.925	7.5	0.847	1.000	0.64	35.1
		SCR	0.950	5.0	0.885	1.000	1.56	36.0
	PS	DCR	1.000	0.0	NA	NA	NA	NA
		SCR	1.000	0.0	NA	NA	NA	NA
	SC	DCR	0.950	5.0	0.885	1.000	0.49	36.0
		SCR	0.975	2.5	0.928	1.000	2.03	36.0
	Fr	DCR	0.975	2.5	0.928	1.000	0.99	35.7
		SCR	0.975	2.5	0.928	1.000	1.01	36.0
Group 2	CM	DCR	0.900	10.0	0.812	0.998	0.29	34.8
		SCR	0.975	2.5	0.928	1.000	3.47	35.7
	CMD	DCR	0.875	12.5	0.778	0.984	0.25	34.5
		SCR	0.975	2.5	0.928	1.000	4.00	35.7
	MI	DCR	0,875	12,5	0,778	0,984	0,59	35,7
		SCR	0.925	7.5	0.847	1.000	1.69	35.7
	ST	DCR	0.900	10.0	0.812	0.998	0.71	35.1
		SCR	0.925	7.5	0.847	1.000	1.40	36.0
	PS	DCR	0.950	5.0	0.885	1.000	0.50	35.7
		SCR	0.975	2.5	0.928	1.000	1.99	36.0
	SC	DCR	0.900	10.0	0.812	0.998	0.28	35.7
		SCR	0.975	2.5	0.928	1.000	3.52	36.0
	Fr	DCR	0.900	10.0	0.812	0.998	0.28	35.4
		SCR	0.975	2.5	0.928	1.000	3.52	36.0

Here and in the following tables: CM—color match, CMD—cavosurface marginal discoloration, MI—marginal integrity, ST—surface texture, PS—postoperative sensitivity, SC—secondary caries, Fr—fracture, DCR—direct composite restoration, SCR—strip crown restorations, NA—it is impossible to make calculations because no events occurred during the entire observation period.

**Table 3:** Results of a log-rank test for comparing long-term results after DCR between two groups: those with the absence of biological factors of caries risk (Group 1) and the presence of biological factors of caries risk (Group 2).

	Criterion	Chi-Square Test	*p*-Value	Power	Sample Size
Group 1 vs. Group 2	CM	0.0	1.0	0.025	>1000
	CMD	0.1	0.7	0.051	>1000
	MI	1.3	0.2	0.177	454
	ST	0.1	0.7	0.055	>1000
	PS	2.0	0.2	0.210	316
	SC	0.7	0.4	0.114	880
	Fr	1.9	0.2	0.216	336

Here and in the following tables: *p* > 0.10 No evidence against the null hypothesis.

**Table 4:** Results of a log-rank test for comparing long-term results after SCR between two groups: those with the absence of biological factors of caries risk (Group 1) and the presence of biological factors of caries risk (Group 2).

	Criterion	Chi-Square Test	*p*-Value	Power	Sample Size
Group 1 vs. Group 2	CM	0.0	1.0	0.025	>1000
	CMD	0.0	1.0	0.025	>1000
	MI	0.2	0.7	0.062	>1000
	ST	0.2	0.6	0.062	>1000
	PS	1.0	0.3	0.126	628
	SC	0.0	1.0	0.025	>1000
	Fr	0.0	1.0	0.025	>1000

**Table 5:** The results of a Log-rank test for comparing long-term results in a group without any biological factors of caries risk (Group 1) between DCR and SCR restoration techniques.

	Criterion	Chi-Square Test	*p*-Value	Power	Sample Size
DCR vs. SCR	CM	1.9	0.2	0,216	336
	CMD	1.9	0.2	0,216	336
	MI	0.0	1.0	0,025	>1000
	ST	0.2	0.6	0,062	>1000
	PS	NA	NA	NA	NA
	SC	0.3	0.6	0.076	>1000
	Fr	0.0	1.0	0.025	>1000

**Table 6:** The results of a log-rank test for comparing long-term results in a group with biological factors of caries risk (Group 2) between DCR and SCR restoration techniques.

	Criterion	Chi-Square Test	*p*-Value	Power	Sample Size
DCR vs. SCR	CM	1.9	0.2	0.216	336
	CMD	2.8	0.1	0.294	226
	MI	0.5	0.5	0.099	>1000
	ST	0.2	0.7	0.055	>1000
	PS	0.4	0.6	0.076	>1000
	SC	1.9	0.2	0.216	336
	Fr	1.9	0.2	0.216	336

## Data Availability

Not applicable.
